# A New Regulating Device

**Published:** 1892-10

**Authors:** Edw. C. Kirk


### ARTICLE V.

### A NEW REGULATING DEVICE.

BY EDW. C. KIRK, D. D. S.

The following contribution to the list of devices for correcting one of the most frequently occurring forms of dental irregularity may be found useful. I have obtained very satisfactory results with it in the two cases where I have used it. The object to be accomplished in both of these was to move the incisors outward for the purpose, in the first instance, of increasing the size of the upper arch, and in the second instance, where the upper central incisors closed inside of the lower incisors, of correcting this defect.

This appliance is constructed as follows: A broad clasp of platinized gold is thrown around each of the sixth-year molars, the opening of the clasp being at the distal buccal angle of the tooth or on its distal proximal surface These clasps are yoked together by a narrow silver plate simply to give solidity to the fixture and cause the two molars to act as a single abutment. On the buccal aspects of each molar clasp is soldered a section if gold tubing about three-eighth of an inch in length; the tubing used is the hinge tubing of watch-case makers. The distal end of each tube is closed with a drop of gold solder. When the appliance is thus far completed, it is placed on the model and a section of piano wire bent to conform to the arch, impinging on the baccal aspect of the teeth about the middle of their crowns. The length of the piano-wire spring and the relation of its curvature to the labial aspects of the incisors is made such that its form will be that which the arch of the anterior teeth are to take and permanently retain, or, in other words, the wire is to be bent to the form of the arch which it is desired the teeth shall form when the correction is completed.

The wire will now have proximately a U-shape. The free extremities of the wire are to be cut to the proper length, so that when introduced into the tube-sockets with the fixture in position in the mouth, the arch of the U will stand nearly one-quarter of an inch anterior to the incisors and about the middle of their crowns. After the fixture is adjusted in the mouth, the teeth to be moved outward are to be firmly ligated to the piano wire by means of fine gilling-twine or silk. The especial advantages of this fixture are that it is cleanly and easily removable for readjustment or cleansing. The traction force is exerted directly outward in the median line, or, if desired, its direction may be modified by tying the ligature to the wire on either side of the tooth to be moved. The amount of force exerted is absolutely under control, and may be modified at any time by changing the length of the legs of the U-shaped wire, or bv using a wire of smaller diameter, or by the method of tying the ligature to the wire.

Not the least important advantage of the appliance as described is, that by having proper regard for the curve of the spring and length of its sides in relation to the curvature of the corrected arch, the fixture becomes practically automatic from the fact that the limit of resilience of the spring is reached at the moment the irregularity is corrected. Consequently, when the work of correction is done, the spring ceases to act. I have found much satisfaction in the use of electro-gilded piano wire, not only in the cases described, but wherever I have occasion to use piano wire in regulating teeth. The deposit of gold is amply sufficient to protect the wire from oxidation during a reasonable time while in use in the mouth, and the unsightly staining of the teeth by iron salts is thus avoided. The gold surface is also an advantage when it becomes necessary to unite lugs, loops, or other fixtures to it by means of solder.—*Cosmos*.

---

ARTICLE VI.

